# Follicle development as an orchestrated signaling network in a 3D organoid

**DOI:** 10.1186/s13036-018-0134-3

**Published:** 2019-01-09

**Authors:** Andrea S. K. Jones, Ariella Shikanov

**Affiliations:** Department of Biomedical Engineering, University of Michigan, 2126 Lurie Biomedical Engineering, 1101 Beal Avenue, Ann Arbor, MI 48109 USA

**Keywords:** Ovarian folliculogenesis, Crosstalk, Cytokines, 3D culture, Biomaterials

## Abstract

The ovarian follicle is the structural and functional unit of the ovary, composed of the female gamete (the oocyte) and supportive somatic cells. Follicles are not only the source of a female’s germ cell supply, but also secrete important hormones necessary for proper endocrine function. Folliculogenesis, the growth and maturation of the follicular unit, is a complex process governed by both intrafollicular crosstalk and pituitary-secreted hormones. While the later stages of this process are gonadotropin-dependent, early folliculogenesis appears to be controlled by the ovarian microenvironment and intrafollicular paracrine and autocrine signaling. In vitro follicle culture remains challenging because of the limited knowledge of growth factors and other cytokines influencing early follicle growth. Here we discuss the current state of knowledge on paracrine and autocrine signaling influencing primary follicles as they develop into the antral stage. Given the importance of intrafollicular signaling and the ovarian microenvironment, we reviewed the current engineering approaches for in vitro follicle culture, including 3D systems using natural hydrogels such as alginate and synthetic hydrogels such as poly(ethylene glycol). Our discussion is focused on what drives the proliferation of granulosa cells, development of the thecal layer, and antrum formation—three processes integral to follicle growth up to the antral stage. Further research in this area may reveal the mechanisms behind these complex signaling relationships within the follicle, leading to more successful and physiologically-relevant in vitro culture methods that will translate well to clinical applications.

## Background

Ovarian folliculogenesis encompasses a wide breadth of cellular processes beginning in the dormant, primordial follicle and culminating in a fully mature and developmentally competent oocyte that is ovulated and becomes available for fertilization. This highly ordered process is influenced by a milieu of factors from various physiological domains, particularly the hypothalamic-pituitary-gonadal (HPG) axis. The HPG axis governs the secretion of hormones that cause cyclical changes in the reproductive organs. The secreted hormones, beginning with gonadotropin-releasing hormone (GnRH) from the hypothalamus, as well as downstream hormones follicle-stimulating hormone (FSH), luteinizing hormone (LH), estrogen, progesterone, and others, become particularly important during the preovulatory stage of folliculogenesis, after the follicle’s antrum has formed and the oocyte has matured. However earlier stages of folliculogenesis can proceed independent of gonadotropins and are instead dependent on intrafollicular signaling between the oocyte and the somatic cells present. New studies continue to emerge revealing the importance of extracellular signaling factors within the follicle microenvironment and elucidating the mechanisms by which intra- and inter-follicular cytokines initiate and sustain complex paracrine and autocrine signaling relationships that govern the various processes of folliculogenesis until gonadotropins gain predominate influence. The complexity of intrafollicular crosstalk can be attributed in part to the follicle’s 3D architecture and the juxtaposition of the cells within the follicle. As the follicle expands throughout folliculogenesis, the somatic cells acquire distinct phenotypic characteristics due to the gradient that develops via diffusion of systemic factors into the follicle and oocyte-derived factors outward from the oocyte. The importance of these gradients in developing cumulus and mural granulosa cell lineage have been previously studied and there are perhaps many other processes influenced by these intrafollicular gradients that have yet to be uncovered [1].

**Fig. 1:**
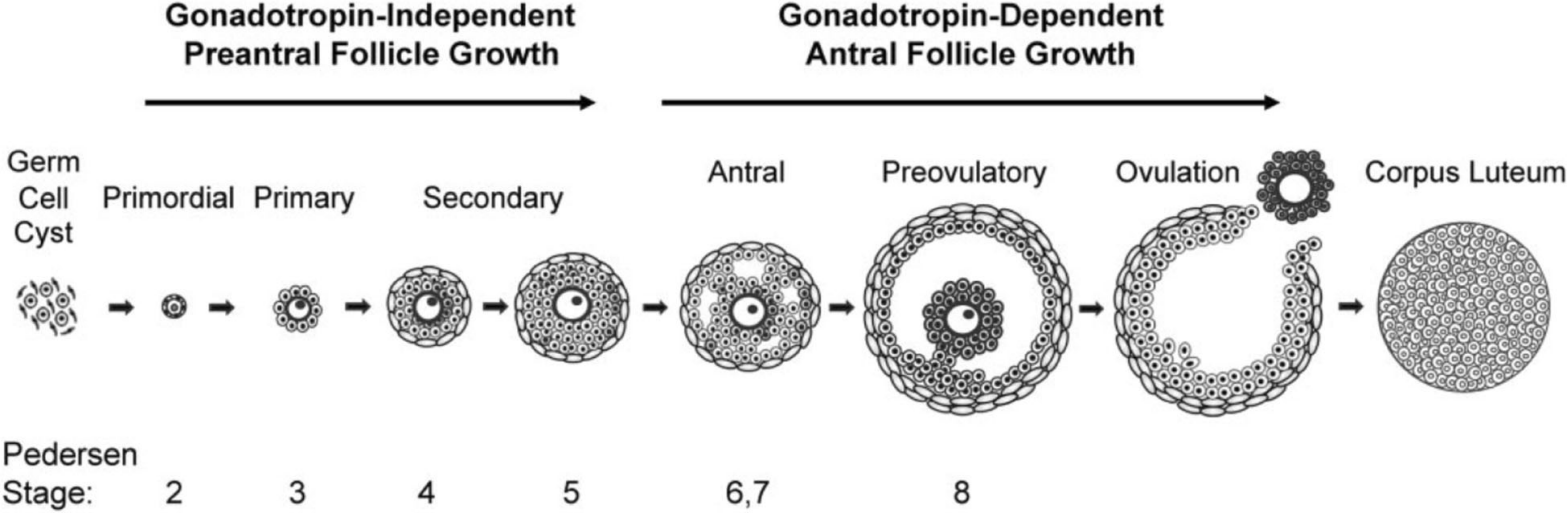
Schematic detailing the stages of mammalian folliculogenesis taken from Edson et al., “The mammalian ovary from genesis to revelation,” Endocrine Reviews, 2009, 30, 6, pp. 624–712 by permission of Oxford University Press [2]

The first stage of folliculogenesis begins when the primordial follicle, composed of an oocyte and single layer of pre-granulosa cells, is activated [2]. This process is not well understood but oocyte-specific transcription factors such as newborn ovary homeobox (NOBOX), spermatogenesis and oogenesis helix-loop-helix 1 (SOHLH1), and spermatogenesis and oogenesis helix-loop-helix 2 (SOHLH2) are believed to be critical to this initial step [2–5]. At this point, the oocyte is arrested in the diplotene stage of prophase I of meiosis and will not regain meiotic competence until just before ovulation [6]. The follicle transitions into its primary state as the pre-granulosa cells transform from a squamous to cuboidal morphology and begin to proliferate. These granulosa cells are surrounded by a layer of extracellular matrix called the basement membrane that acts as a permeable barrier between the follicle and its environment. The secondary stage begins as the follicle acquires multiple layers of granulosa cells and the thecal layer begins to form outside the basement membrane. The theca layer will also develop vasculature to provide nutrients as the follicle expands; however this vasculature does not penetrate the basement membrane and most soluble factors diffuse in and out, contributing to the intrafollicular gradient. During this period the oocyte grows in size but remains in meiotic arrest. However the cytoplasmic maturation of the oocyte that takes place during folliculogenesis has been shown to be equally important to meiotic competency for successful in vitro maturation, meaning that the oocyte must reach a sufficient diameter and accumulate the nutrients necessary for early embryonic development [7]. The antrum forms as a result of both granulosa cell secretions and fluid from the thecal vasculature. Before ovulation, a surge of LH causes the oocyte to mature further, resume meiosis and progress to metaphase II [8]. The granulosa cells transition again into cumulus cells and respond to the LH surge by secreting hyaluronic acid in a process called cumulus expansion [8]. Following ovulation, the remaining granulosa and theca cells form the corpus luteum, which secretes progesterone and inhibin—key hormones to sustain the earliest stages of pregnancy. This structure degenerates in a matter of days if pregnancy does not result, and the cycle initiates again. Follicles are normally classified with names based on functional stage but can also be categoriezed according to the Pedersen and Peters system [9]. This process, along with common follicle classifications, is visually depicted in Fig. 1, taken from Edson, Nagaraja, and Matzuk (2009).

The importance of crosstalk between the oocyte and its surrounding somatic cells has long been recognized and was first characterized by Gregory Pincus and E.V. Enzmann in 1935. Their study involved the isolation of mature oocytes from the follicular structure, upon which they observed spontaneous resumption of meiosis, leading them to conclude that the cells surrounding the oocyte somehow held it in meiotic arrest [10]. This observation has inspired numerous researchers since, who have identified some of the particular mechanisms by which the follicular structure regulates its own growth and maturation. Later studies revealed how the follicle’s somatic cells temporally promote oocyte maturation via various paracrine signals and even more recently, multiple research groups have identified and characterized numerous factors secreted by the oocyte that influence granulosa and theca cell processes [8, 11–13] . The characterization of these signaling pathways is opening doors for researchers in diverse areas of reproductive science where this information can be applied in the laboratory and in the clinic.

Here we will discuss the state of knowledge in paracrine signaling governing the primary-to-antral follicle transition, specifically in murine models that are often used in the laboratory setting. Studies using other animal species, such as bovine and ovine models were reviewed elsewhere [8, 14–17]. The primary-to-antral period of growth, characterized by the proliferation of granulosa cells, formation of the thecal layer, and antrum formation, among other processes, is of especial interest to scientists and engineers designing in vitro follicle culture systems to promote folliculogenesis and obtain fertilizable eggs. While progress has been made and promising new culture techniques are emerging, feasible and reproducible culture methods to grow and mature early stage follicles in a dish are still difficult to attain. Researchers have also struggled to leverage the ample supply of primordial follicles in the ovary, as some of the particular mechanisms of early stage folliculogenesis have yet to be uncovered. We will conclude our discussion by highlighting some of the most recent models implemented for in vitro follicle culture that maintain the follicle’s 3D architecture and how these methods can be applied to the growing body of knowledge on folliculogenesis.

### Granulosa cell proliferation

**Fig. 2:**
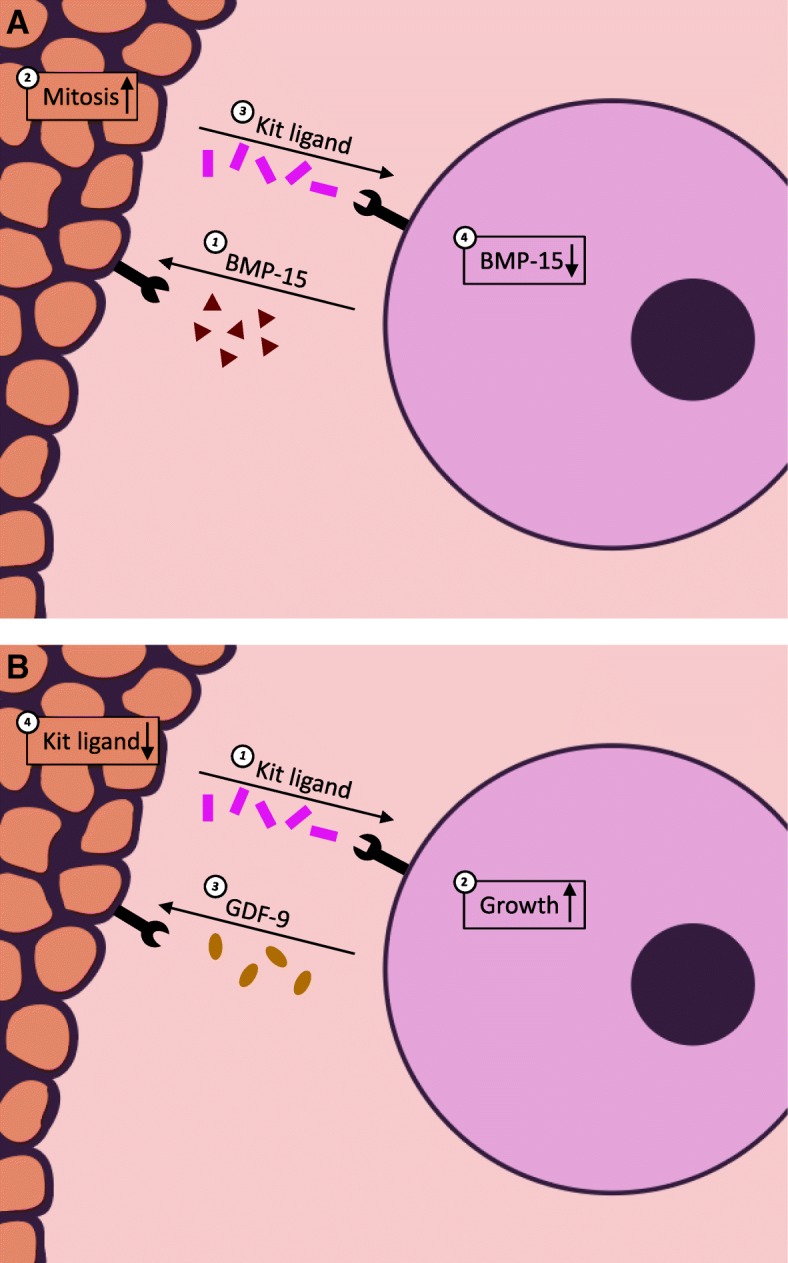
**a**) Oocyte-secreted BMP-15 promotes mitosis and kit ligand expression in granulosa cells and **b**) granulosa cell-secreted kit ligand promotes oocyte growth but is suppressed by oocyte-secreted GDF-9

During the late primary stage of folliculogenesis, a phenotypic change occurs in granulosa cells and they become proliferative, forming multiple layers around the oocyte. Many cell-secreted factors have been found to influence this process, predominately oocyte-secreted factors: bone morphogenetic protein 15 (BMP-15) and growth differentiation factor-9 (GDF-9) [18, 19]. The influence of BMP-15 on granulosa cell proliferation is particularly interesting and has been well-characterized by various research groups [13, 20–24]. These studies suggest a feedback loop between the granulosa cells and the oocyte involving BMP-15 from the oocyte, kit ligand from the granulosa cells, and c-Kit (the kit ligand receptor), which is expressed in the oocyte but not in granulosa cells. This hypothesized loop is initiated when BMP-15 from the oocyte stimulates the expression of kit ligand in granulosa cells, which binds the c-Kit surface receptor on the oocyte [9]. Kit ligand has been shown to promote oocyte growth and cytoplasmic maturation, though the specific mechanisms by which these changes occur have yet to be uncovered [25–27]. When binding c-Kit, the ligand also inhibits further BMP-15 expression, slowing BMP-15-induced proliferation in the granulosa cells [20]. Once binding bone morphogenetic protein receptor type-1B (BMPRIB/ALK-6) or bone morphogenetic protein receptor II (BMPRII), BMP-15 initiates kit ligand expression in the granulosa cells via activation of the Smad1/5/8 pathway [23]. An illustration of this relationship can be seen in Fig. 2a. BMP-15 appears to be able to induce proliferation independent of FSH activity, indicating its importance in the stages of folliculogenesis preceding gonadotropin dependence [13]. BMP-15 has also been shown to inhibit FSH-induced cytodifferentiation (among other effects) in granulosa cells by inhibiting FSH receptor expression [28]. Together, these findings suggest that BMP-15 impacts both granulosa cell proliferation and FSH-dependent cytodifferentiation, two mechanisms by which the oocyte may direct early follicle growth [28].

GDF-9 has been shown to be necessary for granulosa cell proliferation beyond the primary follicular stage [29–33]. Unlike BMP-15, GDF-9 causes transcriptional changes by binding the transforming growth factor-β receptor 1 (TGFβR1/ALK-5) or BMPRII receptors, causing activation of Smad2/3 on the target cell surface [34]. One study found that this factor induces expression of hyaluronan synthase 2 (HAS2), cyclooxygenase 2 (COX-2), and steroidogenic acute regulator protein (StAR) mRNA in granulosa cells, all of which are key enzymes involved in proliferation [35]. Much like BMP-15, GDF-9 has been implicated to participate in a feedback loop between granulosa cells and the oocyte: kit ligand from the granulosa cells stimulates oocyte growth until a certain threshold is reached, at which point GDF-9 from the oocyte suppresses further kit ligand expression in the granulosa cells, as seen in Fig. 2b [8]. Supporting this hypothesis, one study used Gdf9-null mice to show increased expression of the *Kitl* gene in granulosa cells [36]. Taken together, these pathways may be mechanisms by which the oocyte can initiate, modulate, and terminate follicle growth and maturation [13, 28].

Other cytokines have been shown to modulate granulosa cell proliferation, however the mechanisms behind their impact are not yet characterized. Fibroblast growth factor-8 (FGF-8) has been found to be expressed throughout the follicle in bovine models and specifically in the oocyte in rats [37, 38]. Given its similarity to other members of the fibroblast growth factor family that stimulate granulosa cell proliferation, and one study in which transgenic mice with overexpression of FGF-8 showed ovarian stromal cell hyperplasia, future studies may show this factor to play a role in granulosa cell proliferation [18, 39]. Fibroblast growth factor-2 (FGF-2) or basic fibroblast growth factor (bFGF), secreted by both the oocyte and granulosa cells, has been shown to contribute to granulosa cell proliferation in both bovine and hamster models, and also prevents granulosa cell apoptosis in rats via control over intracellular calcium levels [18, 40–44]. This is not surprising given the proliferative effects of FGF-2 in various types of tissue, however further studies will be necessary to elucidate the mechanism behind FGF-2’s effect on granulosa cells and its concentration in the follicle microenvironment in vivo [18]. Bone morphogenetic protein-6 (BMP-6), secreted by the oocyte, was long suspected to play a role in granulosa cell proliferation due to its upregulation starting at the secondary stage of growth, however this factor does not appear to impact this process [28]. Bone morphogenetic protein-7 (BMP-7), expressed by theca cells, also promotes granulosa cell mitosis, as shown by one study reporting enhanced granulosa cell DNA synthesis and proliferation following BMP-7 treatment in vitro [45]. Theca cells also secrete bone morphogenetic protein-2 (BMP-2), which has been shown to influence granulosa cell proliferation in bovine models but have not been explored in murine models [13, 29]. Various signaling pathways initiated by multiple cytokines have also been shown to be necessary for proper granulosa cell proliferation, including the Hedgehog signaling pathway, the Notch signaling pathway, the canonical Wnt/β-catenin pathway via R-spondin2 (RSPO2) expression, and possibly the Hippo signaling pathway, although there are conflicting conclusions drawn from studies in this last area [46–54].

Granulosa cell proliferation also depends on autocrine signaling. Granulosa cells secrete activin, bone morphogenetic protein-5 (BMP-5), and BMP-2 to promote proliferation [29]. They also secrete epidermal growth factor (EGF) and FGF-8 that lead to increased kit ligand expression, promoting not only their own proliferation but also theca cell proliferation [17, 27, 55]. Migration inhibitory factor (MIF) is expressed by both the granulosa cells and local macrophages and may also influence this process, given that one study showed that anti-MIF antibody inhibited granulosa and theca cell proliferation [17, 56]. Activin A may be another potential factor, as it has been shown to increase granulosa cell proliferation when administered exogenously and has been shown to be present in the preantral follicle microenvironment [57–60]. Interestingly, activin may also have a role in the follicle’s transition from dependence on paracrine/autocrine signals to gonadotropins, as indicated by one study in which activin upregulated FSH receptor expression in undifferentiated granulosa cells [61–63]. Anti-Mullerian hormone (AMH) is also secreted by the granulosa cell population and may play a role in proliferation. This molecule is secreted by the pre-granulosa cells of activated primordial follicles and is more commonly recognized for its potential role in limiting the pool of recruited follicles during each ovulation cycle [64]. However, one study using rat granulosa cells cultured in vitro found that exogenous AMH caused reduction of aromatase and LH receptor expression, and therefore dampened the proliferative effects of FSH [65, 66]. Transforming growth factor- β (TGF-β) may also play a role in granulosa cell proliferation and is secreted by all three major follicular cell types (the oocyte, granulosa cells, and theca cells), however the results of various studies on TGF-β have been inconclusive and have varied across species [17, 29]. One study in rats indicate that theca-secreted TGF-β_1_ may increase granulosa cell production of connective tissue growth factor (CTGF), which may in turn influence the angiogenesis and matrix remodeling necessary for antrum formation, which will be discussed in detail later [67].

It should also be noted that many of these secreted factors play equally important roles in regulating steroidogenesis in the later stages of folliculogenesis, as discussed in previous reviews [8, 13]. In addition to further studies using transgenic rodents and in vitro culture, gene expression analysis and transcriptome studies, such as a recent study characterizing transcriptional regulation between the oocyte and granulosa cells, must also be performed to fully understand this and other follicular processes [68].

### Theca layer development

As the follicle progresses towards a gonadotropin-dependent state, the theca layer begins to form. This layer is comprised of cells largely believed to be recruited from the ovarian stroma, possibly of a fibroblast-like origin [69]. This recruitment is most likely mediated by an oocyte- or granulosa cell-secreted factor, however this factor or group of factors have not been identified or characterized [27, 70]. The theca layer of cells not only provides structural support for the follicle but secretes signals relevant to oocyte-granulosa cell crosstalk and produces key gonadotropins in later stages of folliculogenesis. These cells also become a major constituent of the corpus luteum after ovulation, where they continue to serve their sex hormone-producing purposes [70]. Two distinct populations of theca cells emerge as the secondary stage of folliculogenesis begins: a theca externa, expressing bone morphogenetic protein-4 (BMP-4), and a theca interna expressing BMP-7 [71]. The theca interna becomes highly vascularized as folliculogenesis progresses, while the theca externa is composed of a protective fibrous tissue [72].

Many of the oocyte- and granulosa cell-secreted factors previously discussed also regulate development of the theca layer. Kit ligand from granulosa cells, secreted in part due to oocyte-secreted GDF-9 and BMP-15, leads to theca cell proliferation via the Erk1/2 pathway [21, 27, 55]. One study using rat theca cells showed increased differentiation and androsterone production when cells were treated with kit ligand and insulin-like growth factor 1 (IGF-1), another granulosa cell-secreted factor [73]. GDF-9 also plays a key role in proper thecal layer development, as demonstrated by one study using a GDF-9 deficient mouse model [36]. Important thecal markers were not detectable and a distinct layer could not be observed using light or electron microscopy, indicating that without GDF-9, the follicle was unable to recruit thecal precursor cells [36]. Whether GDF-9 affects the thecal layer directly or indirectly is unknown, however this study suggests some secreted thecal precursor recruitment factor is modulated by GDF-9 expression, indicating an indirect influence [36]. Granulosa cell- and macrophage-secreted MIF may also influence thecal development, as previously described [56]. Fibroblast growth factor-7 (FGF-7) may also contribute to early recruitment and development of the thecal layer, as indicated by one study in which the factor was found localized in thecal precursor cells [74]. This research group hypothesized that FGF-7 production in these cells was stimulated by kit ligand from the granulosa cell population and created a positive feedback loop in which FGF-7 upregulates kit ligand production [74]. Granulosa cell-derived EGF and FGF-8 also upregulate theca layer development, as previously mentioned.

As the theca layer develops, angiogenesis occurs such that new capillaries become integrated into the layer. This process can be modulated by granulosa cell-secreted FGF-2 and circulating leptin, platelet-derived growth factor subunit B (PDGFB), and vascular endothelial growth factor (VEGF) already present in the follicle [17, 18, 72, 75, 76]. VEGF expression in the granulosa and theca cells increases as folliculogenesis continues and contributes to the increasing vascularization and oxygenation of the follicle [77]. VEGF expression in these cell types can be mediated by interleukin 6 (IL-6), FSH, and human chorionic gonadotropin (hCG) [77].

### Antrum formation

The formation of the fluid-filled antrum cavity marks an important transition in follicle development. During preantral growth, multiple pockets of fluid form throughout the follicle and then coalesce into one large cavity [78]. As the antrum forms, the granulosa cells are divided into two populations: the cumulus granulosa cells proximal to the oocyte and the mural granulosa cells lining the inside of the follicular wall [79]. The follicular fluid in this cavity is composed largely of components from the blood that diffuse out of the thecal capillaries, as well as secretions from the granulosa cells like hyaluronan [78]. Researchers hypothesize that this process largely depends on the development of an osmotic gradient that draws fluid into the follicle from the thecal vasculature. Aquaporins, transmembrane proteins that serve as water channels, are expressed by the granulosa cells and may help to facilitate this process via the influx of fluid from the theca layer’s vasculature that coalesces into the antral structure [53, 80]. Granulosa cell secretions of hyaluronan, a glycosaminoglycan largely found in extracellular matrix throughout the body’s tissues, and versican, an extracellular matrix proteoglycan, are believed to contribute to this process, as previously mentioned [78]. Versican may also bind hyaluronan molecules in order to keep them within the antrum [78].

Antrum formation is predominantly influenced by FSH secreted by the pituitary, however some paracrine signals have been shown to exert indirect influence over this process [27]. In one study, preantral follicles were stimulated with activin A and showed increased formation of antrum-like structures [57]. In fact, in this study antrum formation did not occur without the addition of activin A to the culture medium, even when FSH was present [57]. Like the other processes discussed thus far, kit ligand from the granulosa cells is necessary for antrum formation as the follicle progresses towards ovulation, perhaps partly due to its ability to stimulate somatic cell proliferation and other related processes that affect antrum formation downstream [21, 26, 78, 81, 82]. In one study, the monoclonal anti-c-kit antibody ACK_2_, which blocks kit-kit ligand binding, was injected in vivo and suppressed antrum formation [83]. Numerous factors have been shown to influence the secretion of versican by the granulosa cells, including LH and hCG, as well as exogenous forskolin, an adenylate cyclase stimulator that increases intracellular cAMP [46]. This study also found versican to be expressed in primary follicles (before FSH influences growth), suggesting that its expression at this early stage might be mediated by a member of the TGFβ family like activin or GDF-9 [46].

Many other oocyte- and somatic cell-secreted factors may influence antrum formation but have yet to be identified. Because angiogenesis plays a large role in the formation of the thecal vasculature, and antrum formation in turn depends on the diffusion of fluid out of the thecal capillaries, angiogenic factors like VEGF and leptin may indirectly influence antrum formation [78]. Similarly, in order for the antrum to form, major extracellular matrix remodeling must occur. As previously mentioned, TGF-β_1_ from theca cells may mediate granulosa cell production of CTGF, a factor known to mediate angiogenic processes and extracellular matrix remodeling [67, 84]. This discussion brings to light the deeply complex signaling relationships governing the gonadotropin-independent stages of folliculogenesis. Table 1 highlights some of the factors discussed that are most well-characterized in murine models. Much is still unknown about how these factors interact and facilitate various processes within the follicular structure. Without this knowledge, researchers lack some of the tools necessary to develop novel engineering methods for in vitro follicle culture and for clinical applications related to various reproductive disorders.

**Table 1:** Follicle-secreted factors and their functions, classified by secreting cell type

Oocyte-Secreted Factors	
BMP-15, GDF-9	Promotes granulosa proliferation and theca layer development [13, 20–24, 27, 35, 55]
FGF-2, FGF-8	Promotes granulosa cell proliferation and survival [37, 39–42, 44]
Granulosa Cell-Secreted Factors	
Activin, BMP-2, BMP-5	Promote granulosa cell proliferation [29, 57–60]
MIF, EGF, FGF-8	Promote theca layer development [17, 27, 55, 56]
FGF-2	Promote theca layer angiogenesis [18, 76]
Aquaporin, Versican	Facilitate antrum development [53, 78, 80]
Theca Cell-Secreted Factors	
BMP-7	Promotes granulosa cell proliferation [45]
FGF-7	Promotes theca layer development [74]

### Existing bioengineering approaches to direct Folliculogenesis

**Fig. 3:**
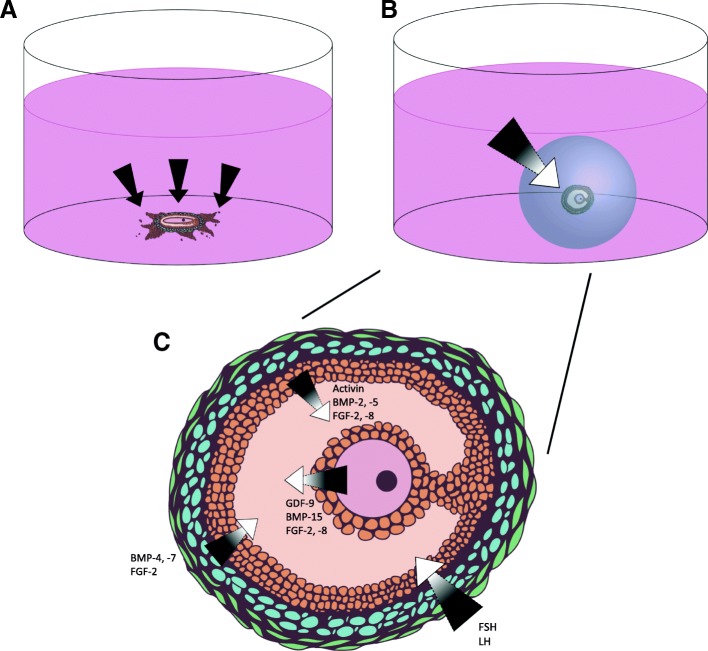
Growth factors and nutrients are more capable of promoting follicle growth when the structure is cultured in 3D (**b**) versus in 2D culture (**a**), especially given the complex crosstalk between cells within the follicle (**c**)

In order to elucidate the unknown mechanisms of folliculogenesis and translate this new knowledge into clinical applications, physiologically-relevant and reproducible in vitro culture systems must be developed for the laboratory setting. John Eppig was the first to culture follicles in vitro in 1977, using a 2D method involving enzymatic digestion of ovarian tissue to collect follicles, followed by culture in 24-well plastic culture dishes [85]. His studies indicated that oocytes can be grown in vitro but require physical contact with their somatic cells [85]. A more recent study from J. Eppig’s group investigating the transcriptional activity in follicles at different developmental stages indicated that as the follicle grows, its 3D structure leads to gradients of nutrients, oxygen, oocyte-derived and systemic factors between the layers of granulosa cells in a follicle. Granulosa cells proximal to the oocyte receive more oocyte-derived factors than those further away, and those further away receive higher concentrations of systemic hormones and paracrine factors [1]. These gradients were shown via gene expression analysis to impact cell differentiation and follicle growth and maturation [1]. Given the importance of maintaining the follicle’s complex architecture, 3D culture methods have become the standard for researchers in this field. A comparison of 2D and 3D culture methods in the context of maintaining intrafollicular signaling gradients can be seen in Fig. 3. Numerous engineering approaches have been employed to meet the mechanical needs of ovarian follicles, however encapsulating follicles in natural or synthetic hydrogels is the most widely used. These microenvironments must meet several design criteria, as previously outlined by Shea, Woodruff, and Shikanov [86]. These include gentle culture conditions, maintenance of cell-cell connectivity, adequate diffusivity for nutrients from culture medium, an allowance for significant follicle expansion, and ease of retrieval upon experiment completion [86].

With regards to providing mechanical stability, alginate was the first biomaterial to be implemented for follicle culture. This polysaccharide is derived from algae and has a mild gelation process ideal for use with follicles [86]. The hydrogel can be modified by immobilizing extracellular matrix proteins or peptides in the gel that will allow the follicle to interact with its microenvironment and can be easily dissolved using alginate lyase. One of the first studies to use alginate for follicle culture reported both oocyte growth and granulosa cell proliferation in these encapsulated follicles [87]. Since this initial study, various research groups have optimized their own methods for follicle culture using alginate and these 3D systems have led to numerous discoveries regarding folliculogenesis. Researchers generally use alginate at lower concentrations, as concentration has been found to be inversely proportional to the follicle’s capacity for antrum formation and maturation [88–91]. Alginate is non-susceptible to mammalian enzyme degradation and subjects encapsulated follicles to constant or increasing compressive forces during culture. Incorporation of fibrin into alginate hydrogels allows cellular proteases to degrade the matrix over time and creates a dynamic mechanical environment. One of the early studies using this hydrogel system reported the successful culture of secondary follicles, resulting in an 82% recovery rate of meiotically competent oocytes at the end of culture [92]. Fibrin-alginate was used in later studies by two research groups to study in vitro maturation of baboon oocytes and in vitro culture of primary and secondary rhesus macaque follicles, respectively [93, 94]. These nonhuman primate studies are important steps towards culture of human follicles in vitro, however naturally-derived matrices come with inherent heterogeneity that may not translate well into clinical applications. Poly(ethylene-glycol) (PEG) has also emerged as a popular hydrogel for follicle culture. This synthetic polymer can be modified with functional groups that result in varied gelation times and mechanical properties [95, 96]. Like many of its naturally-derived counterparts, PEG can be modified with peptides that will allow the follicle to degrade the matrix as it grows [91]. PEG may emerge as a superior hydrogel for in vitro follicle culture as scientists and engineers look towards clinical implementation of their methods.

**Fig. 4:**
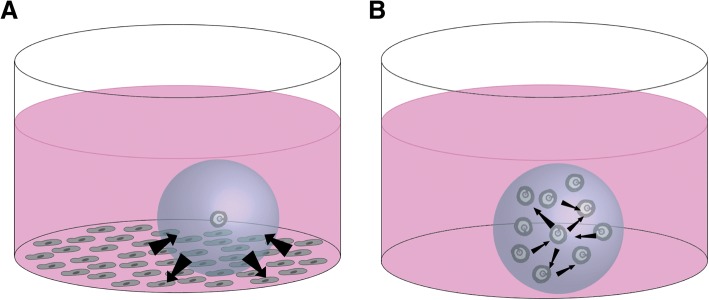
**a**) Co-culture with feeder cells can provide follicles with secreted factors in order to enhance growth and **b**) multiple follicle culture can also enhance growth and development via interfollicular crosstalk

Other research teams have focused on optimizing in vitro culture conditions to recapitulate the complex milieu of growth factors present in vivo. “Feeder cells” have often been employed to provide these secreted factors, as shown in Fig. 4 [91]. Common cell types used in these models include ovarian mesenchymal cells, murine embryonic fibroblasts (MEFs), stromal cells, and granulosa cells [97–100]. One study tested five different cultures systems to elucidate the most successful co-culture conditions for in vitro follicle culture: monoculture in minimum essential medium and in coconut water, as well as co-cultures with ovarian mesenchymal cells, granulosa cells, or cumulus cells [97]. Both monoculture conditions showed little success, however the co-culture conditions showed significantly increased growth rates and oocyte retrieval rate, with co-culture with cumulus cells or mesenchymal cells being most successful [97]. In another study, preantral follicles were cultured with MEFs and researchers observed increased survival (90% vs. 77% in the control group) and significantly larger follicle diameters after 6 and 8 days of culture [98]. In a similar study using MEFs and primary follicles encapsulated in alginate hydrogels, follicles showed increased survival and the majority of oocytes successfully progressed to metaphase II [99]. In this study, fibroblast-conditioned media had similar effects to actual co-culture of follicles with fibroblasts, reinforcing the importance of somatic cell-secreted factors for proper follicle growth [99]. One research group used alginate to encapsulate their co-culture of follicles with ovarian stromal cells, mostly comprised of macrophages and thecal cells [100]. They successfully cultured both late primary and small secondary follicles in their co-culture system and used their results to infer the importance of various secreted factors during early stage folliculogenesis [100].

Multiple follicle culture has also proven an effective method for in vitro culture. Given the improvements to early follicle culture seen using co-culture systems with the cell types discussed previously, it has become clear that generic culture medium supplements do not provide all the nutrients necessary for early folliculogenesis, but some of the factors secreted by other cell types enhance growth. Multiple follicle culture seeks to provide a microenvironment with an amplified supply of follicle-secreted factors that may be necessary for early folliculogenesis via interfollicular crosstalk, as shown in Fig. 4 [101]. Co-culture with other cell types is not a sustainable in vitro culture method, given foreseeable difficulties in identifying media components that can satisfy both cell types; multiple follicle culture, however, may indicate what factors are secreted by follicles and are present in the follicle microenvironment, allowing researchers to leverage this information to modify the supplements used for individual follicle culture. In the first study using this method, follicles were encapsulated in alginate in groups of five and ten [101]. Primary follicles cultured in groups showed enhanced growth and survival, the largest number of follicles together (*n* = 10) yielding the best results [101]. This study provided new insight into the importance of the follicular unit and has prompted other research groups to further probe the mechanisms by which multiple follicle culture enhances growth and development. However, multiple follicle culture may not translate well into clinical applications, given the need to encapsulate follicles of homogeneous size and growth stage and the importance of dominant follicle emergence in human folliculogenesis. One study recently sought to expose how paracrine signaling within the follicle might vary between follicles cultured individually or in groups as previously described [102]. Using Transcriptional Activity CEllular aRray (TRACER) technology, they uncovered unique transcription factor expression signatures in follicles cultured in groups of ten versus in groups of five or individually [102]. NF-κB (nuclear factor kappa-light-chain-enhancer of activated B cells), HIF1 (hypoxia-inducible factor-1), and VEGF-A were among the factors with significantly upregulated expression [102]. Continued studies like this may reveal the interactions of cytokines and transcription factors during different stages of folliculogenesis, aiding in our understanding of how crosstalk within the follicle impacts growth and maturation. Future research in this area will work towards the development of a culture medium that includes all the factors necessary for follicle growth at each stage of development [86]. The applications of this new technology would be endless, ranging from discoveries in basic science to development of clinical treatments for various forms ovarian disorders.

## Conclusions

Folliculogenesis is clearly a highly ordered process with a variety of factors being expressed to varying degrees throughout follicle development. Here we have discussed secreted factors pertinent to the primary to antral stages of follicle growth, given the need to enhance in vitro culture methods for early stage follicles. Oocyte- and somatic cell-secreted factors play important roles in early follicle development and may also impact how gonadotropins affect the follicle during later stages of folliculogenesis. Many signaling relationships between the oocyte and granulosa cells, and granulosa and theca cells, have already been characterized, and there are potentially many more to be uncovered. The oocyte has already been shown to hold immense influence over follicle growth and maturation, given the importance of its secretions of BMP-15 and GDF-9. Granulosa and theca cells also appear to perform specific secretory functions throughout folliculogenesis, regulating somatic cell growth and influencing oocyte maturation. Studies on transcriptional changes and gene expression such as those performed by Zhou et al. (2018) and Biase et al. (2018) will also contribute to a more complete picture of crosstalk within the follicle [68, 102]. To clarify these intricate relationships, physiologically-relevant and highly controllable in vitro systems must be implemented, such as the specially-engineered PEG hydrogels previously discussed. Development and application of these models, coupled with the design of a culture medium including all the key secreted factors necessary for growth at different stages, could be applied to individual follicle culture to access the large primordial follicle pool and open new doors for clinical treatment of various female reproductive disorders.

## Abbreviations

AMH: Anti-Mullerian hormone
bFGF: Basic fibroblast growth factor
BMP-15: Bone morphogenetic protein 15
BMP-2: Bone morphogenetic protein-2
BMP-4: Bone morphogenetic protein-4
BMP-5: Bone morphogenetic protein-5
BMP-6: Bone morphogenetic protein-6
BMP-7: Bone morphogenetic protein-7
BMPRIB/ALK-6: Bone morphogenetic protein receptor type-1B
BMPRII: Bone morphogenetic protein receptor II
COX-2: Cyclooxygenase 2
CTGF: Connective tissue growth factor
EGF: Epidermal growth factor
FGF-2: Fibroblast growth factor-2
FGF-7: Fibroblast growth factor-7
FGF-8: Fibroblast growth factor-8
FSH: Follicle-stimulating hormone
GDF-9: Growth differentiation factor-9
GnRH: Gonadotropin-releasing hormone
HAS2: Hyaluronan synthase 2
hCG: Human chorionic gonadotropin
HIF1: Hypoxia-inducible factor-1
HPG: Hypothalamic-pituitary-gonadal
IGF-1: Insulin-like growth factor 1
IL-6: Interleukin 6
LH: Luteinizing hormone
MEF: Murine embryonic fibroblast
MIF: Migration inhibitory factor
NF-κB: Nuclear factor kappa-light-chain-enhancer of activated B cells
NOBOX: Newborn ovary homeobox
PDGFB: Platelet-derived growth factor subunit B
PEG: Poly(ethylene) glycol
RSPO2: R-spondin2
SOHLH1: Spermatogenesis and oogenesis helix-loop-helix 1
SOHLH2: Spermatogenesis and oogenesis helix-loop-helix 2
StAR: Steroidogenic acute regulator protein
TGF- β: Transforming growth factor-β
TGFβR1/ALK-5: Transforming growth factor-β 1
TRACER: Transcriptional Activity CEllular aRray
VEGF: Vascular endothelial growth factor
